# A comparative study of orphan drug prices in Europe

**DOI:** 10.1080/20016689.2017.1297886

**Published:** 2017-03-29

**Authors:** Katherine Eve Young, Imen Soussi, Michiel Hemels, Mondher Toumi

**Affiliations:** ^a^ Pricing, Reimbursement, Market Access, Creativ-Ceutical, Paris, France; ^b^ Pricing, Reimbursement, Market Access, Creativ-Ceutical, Tunis, Tunisia; ^c^ Market Access EMEA, Horizon Pharma, Utrecht, the Netherlands; ^d^ Public Health Department, Aix-Marseille Université, Université de la Méditerranée, Marseille, France

**Keywords:** Rare diseases, orphan drugs, pricing, treatment cost, Europe

## Abstract

**Background and Objective**: This study assessed price differences by comparing annual treatment costs of similarly available orphan drugs in France, Germany, Italy, Norway, Spain, Sweden, and UK.

**Methods**: Annual treatment costs per drug were calculated using ex-factory prices from IHS POLI and country price databases. The treatment cost in the comparator country was compared to the UK and ratios were analysed. Subanalyses were done on disease areas and UK cost quartiles.

**Results**: 120 orphan drugs were included. Compared to the UK, the average costs were more expensive in France (1.13), Germany (1.11), Italy (1.08), Spain (1.07), and were cheaper in Sweden (0.99) and Norway (0.88). The average ratios offered a restrictive view as ratios were greatly heterogeneous (0.26 to 1.92) which was also seen in the different disease areas. The averaged ratios varied minimally among the cost quartiles which shows that cost differences were similar for the most expensive and least expensive orphan drugs in the UK.

**Conclusions**: Individual orphan drug prices can vary widely across European countries, although on average these differences are relatively minor. This study suggests that in Europe, we may not be able predict which country may have higher or lower prices for orphan drugs.

## Introduction

### Rare disease definition and burden

Rare diseases are rare and serious conditions which are defined in the European Union (EU) as life-threatening or chronically debilitating conditions with a prevalence of no more than five in 10,000 people.[1] In the UK, the National Institute of Health and Clinical Excellence (NICE) in England and Wales has defined ultra-rare diseases as diseases affecting less than 1000 patients in the UK.[2] Orphan drugs are medicines intended for the diagnosis, prevention or treatment of rare diseases.

Rare diseases are usually severe conditions with no or limited choice of therapeutic options, and thus present with a high level of unmet need. In 2007, the EMA estimated that there are 5000–8000 rare diseases affecting between 6% to 8% of the total EU population, amounting to 27 million to 36 million people in the EU.[1] The same report documented that five new diseases are described in the medical literature every week, hence current figures are assumed to be higher. It is estimated that only 1% are currently covered by approved treatments in the EU.[3]

### Price of orphan drugs in the EU

Pricing [4] and accessibility of orphan drugs vary among countries in the EU.[5–8] Although orphan designation and marketing authorisation is at a European level, pricing and reimbursement are on a national level, often driven by health technology assessment (HTA) outcomes and with variable impact from external reference pricing (Table 1).[9] In these HTAs, evidence requirements, pricing and reimbursement decision frameworks, and budget ceilings vary. Also, national responsibilities for price listing and the process in which prices are granted vary widely. As a consequence, prices and levels of access may vary.

**Table 1.** Orphan drugs (OD) HTA, pricing, reimbursement, and market access in major European countries.

Country	HTA specificities for ODs	Pricing of ODs	Reimbursement of ODs
France	Undergo the same HTA procedure as non ODs.[8]	Same pricing procedure as non ODs.[11]	Reimbursement through the standard process
	May benefit from a fast track assessment process reducing timelines from 90 days to 15 days.[10]	Price driven by improvement in actual benefit (IAB)/*Amélioration du Service Médical Rendu* (ASMR)	Driven by actual benefit (AB)/*Service Médical Rendu* (SMR) but more flexibility regarding the strength of supporting evidence in ODs.[10]
	Exemption for health economic evaluation applied to ODs with low budget impact (<€30 million/year for a particular indication).[11]	Usually high priced (ASMR I- III).[11]	Most ODs (98%) are reimbursed.[8]
		Almost all innovative drugs enter the French market as part of a price-volume agreement.[11]	
Germany	For ODs authorised by EMA, benefit is considered proven with market authorisation. The G-BA only determines the extent of additional benefit (minor, considerable, major, or non-quantifiable), the categories ‘no additional benefit’ or ‘less benefit’ are not applicable.[12]	Same pricing procedure as non ODs.[11,^12^]	Reimbursement through the standard process. Full reimbursement for the first year following MA.[11,12]
	The initial assessment for ODs is based on the studies for market authorisation and not on a comparison over an appropriate comparator.[12]	Free pricing during the first year following MA, price is negotiated thereafter between the manufacturer and the Federal Association of Statutory Health Insurance Funds (GKV-SV) based on the added value of the OD decided by G-BA.[11,12]	Thereafter, reimbursement of negotiated price based on initial assessment if the revenue is less than €50million/year.[11,12]
	G-BA and IQWIG assessment criteria tend to be more flexible for ODs.[11]		If revenue is more than €50 million over the previous year, OD is subject to an additional benefit re-assessment vs. current standard of care/appropriate comparator.[11,12]
Italy	Undergo the same HTA procedure as non ODs	Same pricing procedure as non ODs but flexible regarding pricing regulations, clinical data requirements, level of clinical uncertainty, and higher price may be accepted.[8,11,13]	Reimbursement through the standard process
	Evaluated by the Italian Medicines Agency (AIFA) and regional HTAs.[8,11,13]	Price is negotiated between AIFA and the manufacturer, confidential discounts and rebates may also apply for ODs.[11]	Most ODs are distributed through hospital channel.[8,11]
Norway	Undergo the same HTA procedure as non ODs.[10]	Same pricing procedure as non ODs. Free pricing at the manufacturer level but the Norwegian Medicines Agency (NoMA) sets maximum prices at the pharmacy purchasing price (PPP) level for all prescription only medicine via external reference pricing. The pharmacies’ mark-up on the PPP is also regulated.[14]	Reimbursement through the standard process but special consideration may be made for chronic rare diseases.[10]
	Evaluated by the Norwegian Knowledge Centre for the Health Services.[14]		
Spain	Undergo the same HTA procedure as non ODs.[11,13]	Same pricing procedure as non ODs.[11]	Reimbursement through the standard process but more likely to be approved.[13]
	Evaluated by national and regional HTA.[11]	The price is decided on the national level based on cost-effectiveness, budget restrictions, reference pricing, value-based pricing, three years’ sales forecasts and manufacturer’s profit.[11]	Decision made by the regional governments
			Reimbursed through hospital channel or specialist centres.[11]
Sweden	Undergo the same HTA procedure as non ODs however, less robust HTA assessment may be used for ODs.[8,11]	Same pricing procedure as non ODs. Free pricing.[8]	Reimbursement through the standard process.
	Evaluated by the Dental and Pharmaceutical Benefit Agency (TLV).[8,11]		Decisions taken by the Dental and Pharmaceutical Benefits Agency (TLV) and approved by the Public Social Insurance based on cost-effectiveness, human value, need and solidarity.[8,11]
	County Councils (regional HTA agencies) may decide to reimburse ODs independently of TLV recommendation.[8,11]		Several ODs approved for reimbursement despite weak cost-effectiveness data, showing a propensity to accept lower levels of evidence for ODs where limited budget impact is expected.[7]
	No specific HTA criteria applied by the County Councils, decisions are done on case by case basis.[8,11]		
UK	Undergo the same HTA procedure as non ODs but NICE and SMC accepts higher uncertainty regarding clinical evidence requirements and apply adjusted criteria for cost-effectiveness threshold.[11,15]	Same pricing procedure as non ODs. Free pricing but must meet profit control criteria applied also to non ODs.[4]	Reimbursement through the standard process.[11]
	SMC has introduced the Patient and Clinician Engagement (PACE) in additional to the standard HTA framework to support HTA decisions for ODs.[11,16]		NICE bases decision on cost-effectiveness although additional criteria, such as equity may also be considered.[11]
	PACE enables the consideration of added benefits of the drug, from both patient and clinician perspectives that may not be fully captured within the conventional HTA.[16]		SMC bases decision on clinical efficacy and cost-effectiveness.[11]

National pricing regulations are often value-based and the value placed on orphan drugs, as with any intervention, varies per healthcare system. As presented in Table 1, price setting is part of value based assessment in most countries except in Sweden, Norway, and the UK where orphan drugs are freely priced at the manufacturer level but are still subject to indirect regulations and profit control. In addition, each national authority has a different perspective on what constitutes value and willingness to pay for a certain value also varies among countries. Some, e.g. Sweden, may value equity in a sense that all patients deserve treatment and put precedence on products that treat the greatest health need, regardless of the budget impact of the orphan drug, while others, e.g. the UK, value equity by paying a similar price for unit of health production irrespective of the unmet needs, thus looking at maximising health outcomes in the face of budget constraints.[7] Other value drivers may include disease rarity, disease severity, the availability of treatment options, the size of clinical benefit, and incremental cost–effectiveness ratio. The individual drug budget impact is rarely considered despite the high per-patient price due to low patient numbers, attributed to the rareness of the condition, and the drug budget impact is usually low. However, the accumulated budget impact of all orphan drugs in the healthcare system is rising and becoming a significant issue.

These differences in value assessment frameworks reflect two notions of social equity: horizontal equity versus vertical equity. Horizontal equity is defined as the equal treatment of equals.[17] On the other hand, vertical equity is the unequal but equitable treatment of unequals.[17,18] A healthcare system which uses a single cost per QALY threshold for all, like the UK, reflects horizontal equity. A healthcare system which recognises the unique state of patients with rare diseases and that these patients are equally entitled to treatment even if it means foregoing efficiency standards, like France and Sweden, reflects vertical equity. As to which should be prioritised is still an ongoing discussion and the answer may differ per institution.[17] A complex issue to address is that the cost per QALY for most orphan drugs is greater than accepted thresholds.[18]

### Significance and objective of the study

Price is a multifactorial decision and there is currently no European consensus on how the value of orphan drugs is and should be assessed.[19] Thus, orphan drug prices may vary among European countries.

The increasing number of orphan drugs granted marketing authorisation by the EMA has resulted in relevant discussions about high costs and affordability in the wake of the economic crisis and health budget austerity.[3,4,17,20–25]

Orphan drugs have been a highlight of discussions due to their higher price than non-orphan drugs.[23] A 2011 budget impact study in 18 countries in Europe [20] showed that the annual patient cost of commercially available orphan drugs varied between €1251 and €407,631 with a median cost of €32,242 per treatment year per patient. The share of the total pharmaceutical market represented by orphan drugs was predicted to peak from 3.3% in 2010 to 4.6% in 2016, and plateau at 4–5% until 2020, where absolute expenditure will increase, but no faster than the growth of the greater EU pharmaceutical market.[20]

Price comparison studies in Europe are sparse. To improve understanding of this issue, the objective of this study is to assess price differences among countries by comparing the annual cost of treatment per patient of similarly available orphan drugs in seven countries in Europe.

## Methodology

Five steps were implemented in order to compare the annual cost of treatment of orphan drugs in France, Germany, Italy, Norway, Spain, Sweden, and the UK.

### Extraction of orphan drugs from EMA website

Orphan drugs granted market authorisation up to 13 June 2016, including drugs with expired or withdrawn orphan drug designations, were extracted for analysis. If approved for more than one indication, the first EMA indication approved was chosen for inclusion in the analysis. If both indications were approved at the same time, the least prevalent indication was chosen for inclusion.

### Extraction of ex-factory price from IHS POLI database and country-specific database

The IHS POLI database [26] was the primary source of price data. Extraction was dated 14 June 2016. For drugs with no available prices in POLI, available country-specific price databases were used: database of drugs and tariffs (Ameli) [27] for France, British National Formulary (BNF) [28] for the UK, and Farmadati Compendio Farmaceutico Telematico database [29] for Italy.

The earliest price was used for cost calculation as we are interested in the prices at launch and drug prices change over time. An exception to this was when using BNF, where current prices were extracted because price history was not available.

Prices in British pound sterling, Swedish krona, and Norwegian krone, were converted to Euros by applying the respective exchange rates: €1 = £0.72, €1 = 9.09 Swedish Krona, € 1= 9.09 Norwegian Krone. Conversion was done by the IHS database system upon extraction and the same conversion rates were used for BNF prices.

### Calculation of annual treatment cost per patient

We calculated the annual treatment cost per patient in each country for each orphan drug based on the annual treatment dose described in the Summary of Product Characteristics (SmPC). Across all seven countries, the indication and posology are the same. There are differences in the preparation and formulation of drugs across countries but these are minor. As much as possible, the same formulation and preparation per product were used in all countries for ease of comparability.

As dosing of orphan drug treatments may vary according to patient age, weight, disease severity, patient needs, disease progression, or disease complications, assumptions were used during the dose and cost computations.

- Average drug dose for an adult was used unless specifically indicated for use in children. For drugs indicated for both adults and paediatric populations, the pivotal studies described in the European Public Assessment Report (EPAR) were consulted for the average age range of the population included in clinical trials, and dosage and cost computation were done for this specific average patient. For weight adjusted and body surface area (BSA) adjusted treatments, the average weight of an adult was set at 70 kg and the average body surface area was set at 1.73 m^2^. Standard average values for other age intervals were also used.[30]
- If the dose is adjustable based on performance results or an average dose was given, information regarding the average treatment duration and dosage from the EPAR and pivotal studies were used. In the same manner, for cycle-based treatments where the number of cycles varies, the mean number of cycles in the pivotal trials was assumed.
- Treatment duration of 365 days was assumed. For drugs used for less than a year, the costs of the total treatment course were analysed as annual costs.
- For treatments administered as injection or infusion, the nearest full vial size was used. The EPAR was consulted if vials can be stored once opened or should be used within the day. Vial wastage in this sense was taken into consideration.
- If there was an unfinished pack at the end of the year or at the end of a treatment cycle, only a proportion of the price of that pack was accounted for.

### Identification of the reference country for country comparison

Out of the seven countries, the country with the most orphan drugs with available prices was set as the reference country. The UK had the most available price data since drug prices are set by the Department of Health (free pricing), hence the UK was used as the reference country for subsequent analyses.

### Comparison of the annual treatment cost of comparable drugs to the reference country

For each orphan drug that was available in the comparator country and in the UK (reference country), the annual treatment cost in the comparator country was compared to that of the UK and the ratios were computed. The cost ratios represented cost differences between the two countries. With the UK set as 1, a ratio of >1 means that the orphan drug is more expensive than the UK counterpart and a ratio of <1 means the orphan drug is cheaper than the UK counterpart. Ratios were averaged per country and the following analyses were done:

- The averaged ratio was compared between the comparator country and the UK.
- The number of orphan drugs falling under four cost ratio categories was assessed: <0.90 (more than 10% cheaper than the UK), 0.90–1 (up to 10% cheaper than the UK), 1–1.10 (up to 10% more expensive than the UK), and >1.10 (more than 10% more expensive than the UK).
- The averaged ratios were compared when the UK reference list was divided into four quartiles based on the annual treatment costs (quartile 1 includes orphan drugs with the highest annual treatment costs in the UK and quartile 4 includes orphan drugs with the cheapest annual treatment costs in the UK).
- The averaged ratios were compared when the UK reference lists was divided into disease areas: (1) infectious and parasitic diseases; (2) diseases of the blood and the immune system; (3) diseases of the circulatory system; (4) diseases of the nervous system; (5) diseases of the respiratory system; (6) endocrine, nutritional and metabolic diseases; (7) neoplasms; (8) diseases not elsewhere classified. A sub-analysis was done on the top three disease areas with the most available orphan drugs in the UK: (1) neoplasms (N = 38); (2) endocrine, nutritional and metabolic disease (N = 28); (3) disease of the blood and immune system (N = 6) tied with diseases of the circulatory system (N = 6).
- A similar analysis was performed using each single country as the reference country. The results are presented in the supplementary file.

## Results

Ninety-five authorised orphan drugs were identified in the EMA website between 2002 and 2016 and were complemented with 25 drugs with expired or withdrawn orphan drug designations, for a total of 120 (Appendix A found in the supplementary material).

Not all orphan drugs are commercially available in all seven countries and not all commercially available orphan drugs had publicly available prices for analysis. UK had the most number of orphan drugs with available price and was used as the reference country in this study (UK 94 drugs, Italy 83, Germany 68, France 67, Norway 67, Spain 42, Sweden 35).

When the UK reference list was compared to the orphan drugs available in the other six countries, the sample size for analysis were as follows: UK–Italy 76, UK–France 65, UK–Germany 62, UK–Norway 61, UK–Spain 40, UK–Sweden 35. Figures are presented in Table 2.

**Table 2.** Number of comparable orphan drugs analysed per country.

	Number of orphan drugs with available price	Number of orphan drugs compared to the UK (UK = 94)
France	67	65
Germany	68	62
Italy	83	76
Norway	67	61
Spain	42	40
Sweden	35	35

**Figure 1.**
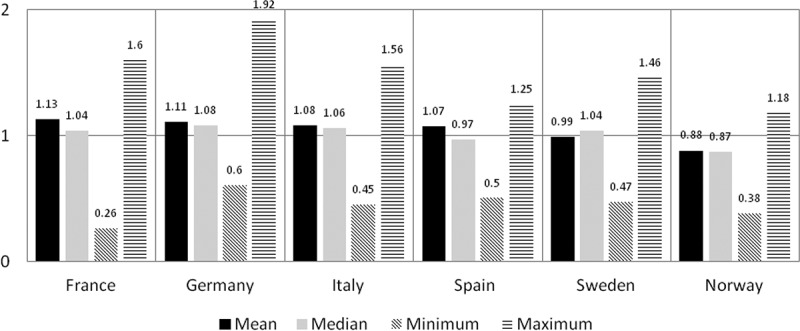
Mean, median, minimum, maximum ratios per country using UK as reference.

Compared to the UK, the average annual costs of orphan drugs were more expensive in France (averaged ratio 1.13), Germany (1.11), Italy (1.08), Spain (1.07), and were cheaper in Sweden (0.99) and Norway (0.88) (Figure 1). The average annual costs ratios varied with the greatest difference at +13%.

The cost differences were greatly heterogeneous and ranged from 0.26 to 1.92, all countries considered (Figure 1). The median ratios ranged from 0.87 to 1.08 (Figure 1).

**Figure 2.**
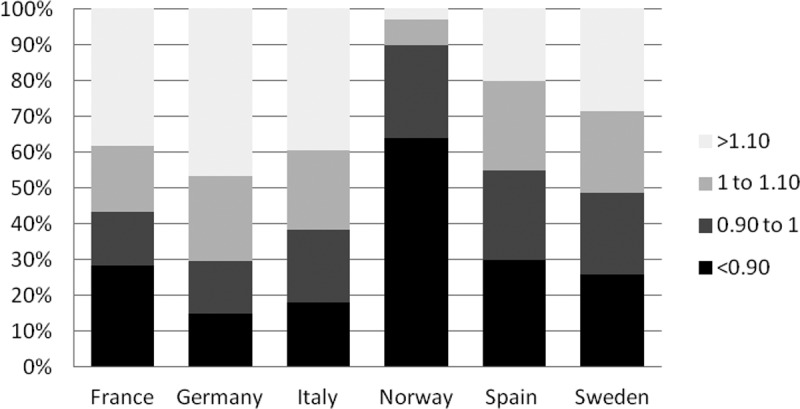
Percentage of orphan drugs (OD) under four cost ratio categories.

Results also showed heterogeneity in cost differences within each country, with a relevant proportion of orphan drugs relatively cheaper or more expensive than the UK (Figure 2). In France, 43% of orphan drugs are cheaper and 57% orphan drugs are more expensive than the UK reference. In Germany, 29% of orphan drugs are cheaper and 71% are more expensive than the UK. In Italy, 38% of orphan drugs are cheaper and 62% are more expensive than the UK. In Spain, 55% of orphan drugs are cheaper and 45% are more expensive than the UK. In Sweden, 49% of orphan drugs are cheaper and 51% are more expensive than the UK. In Norway, 90% of orphan drugs are cheaper compared to the UK.

**Figure 3.**
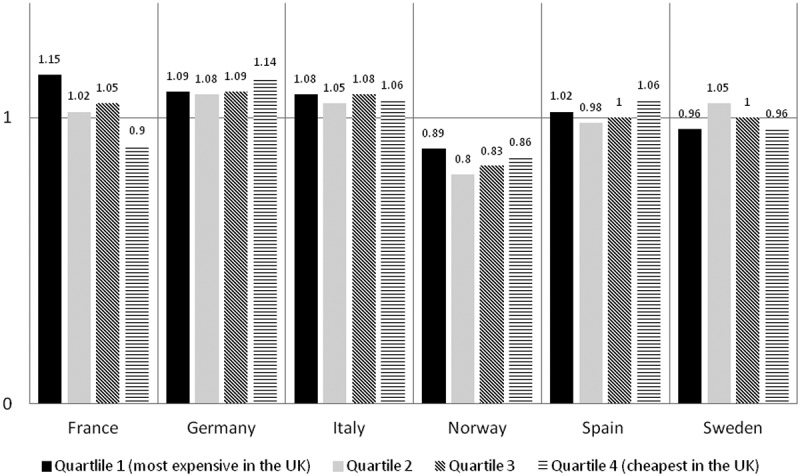
Averaged ratios when the UK reference list was divided into four quartiles based on UK costs. Quartile 1 includes orphan drugs with the highest annual treatment costs in the UK and quartile 4 includes orphan drugs with the cheapest annual treatment costs in the UK.

When we divided the UK orphan drugs into four quartiles based on the most expensive and least expensive, the averaged ratios among the four quartiles varied minimally in all countries except for France (Figure 3).

**Figure 4.**
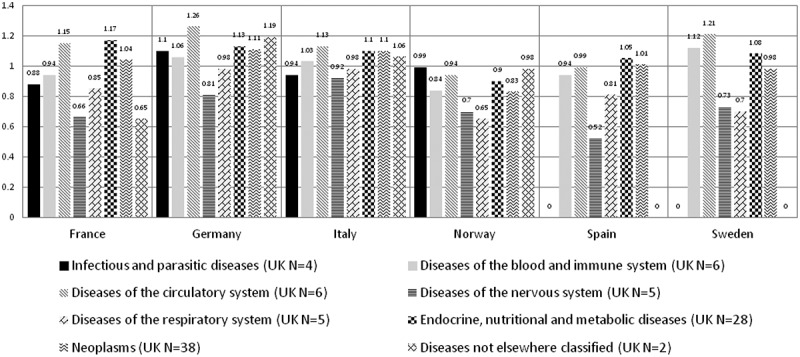
Averaged ratios when the UK reference list was divided into eight disease areas.

Upon looking at the different disease areas, the averaged ratios were heterogeneous in all countries (Figure 4). Orphan drug treatment for diseases of the circulatory system were most expensive than the UK in three of the six countries (Italy, Germany, Sweden) and endocrine, nutritional, and metabolic diseases were most expensive than the UK in two countries (France and Spain) (Figure 4). Treatment for diseases of the nervous system were least expensive than the UK in three of the six countries (Italy, Germany, Spain) and respiratory diseases were least expensive in two countries (Norway and Sweden) (Figure 4).

**Figure 5.**
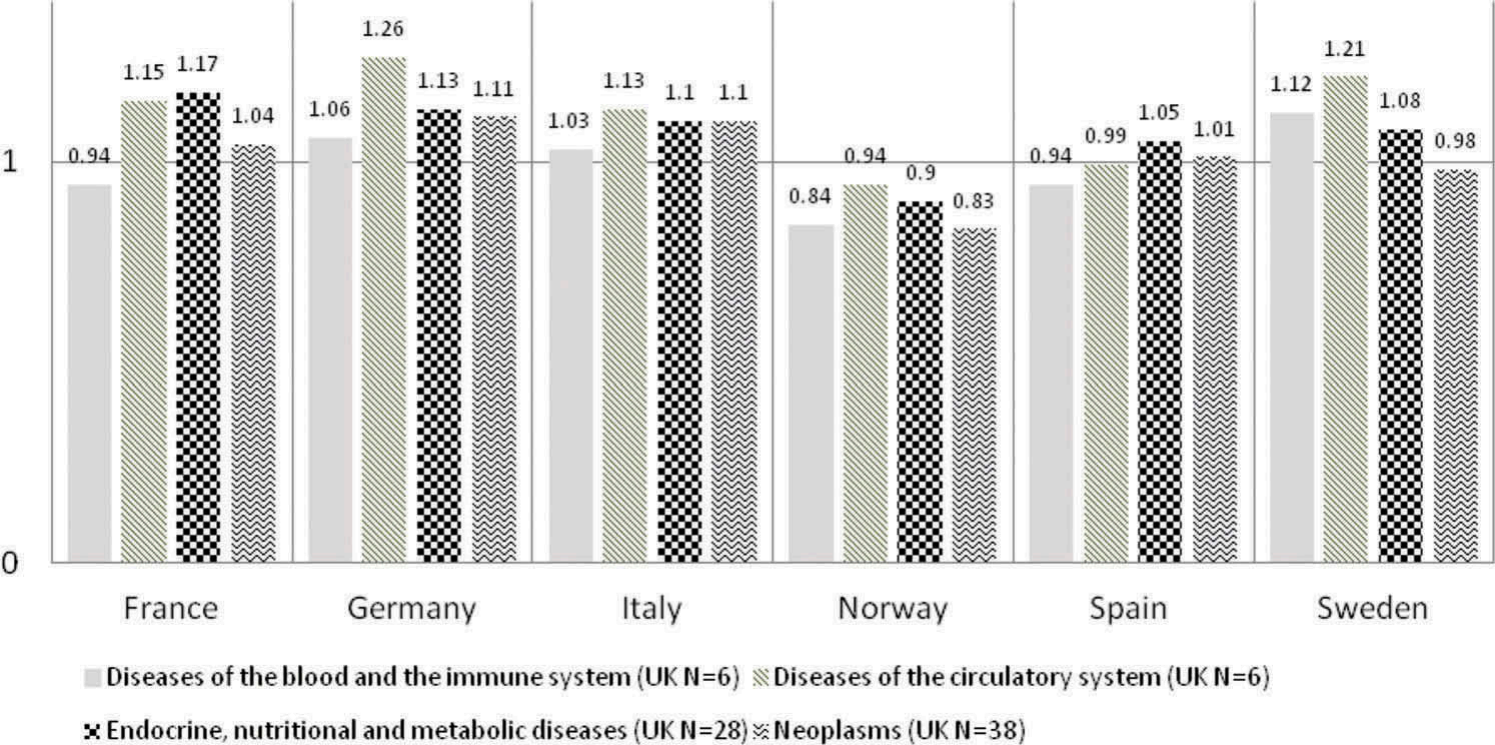
Averaged ratios for top three disease areas with most orphan drugs available in the UK.

Some disease areas had more available orphan drugs than others. The disease areas which had the most orphan drugs in the UK are: (1) neoplasms (N = 38); (2) endocrine, nutritional and metabolic disease (N = 28); (3) disease of the blood and immune system (N = 6) tied with diseases of the circulatory system (N = 6). The averaged ratios among these top disease areas varied minimally in all countries (Figure 5). Of the top disease areas, orphan drug treatments for circulatory disease were most expensive than the UK in four countries (Italy, Germany, Norway, and Sweden) and diseases of the blood and immune system were cheaper than the UK in four countries (Italy, Germany, France, and Spain) (Figure 5).

## Discussion

This study shows that compared to the UK, the average annual treatment costs of orphan drugs were more expensive in France (averaged ratio 1.13), Germany (1.11), Italy (1.08), Spain (1.07), and was cheaper in Sweden (0.99) and Norway (0.88). This finding can be viewed as to be in parallel with the three previous orphan drug pricing studies in Table 3 which showed that most often than not, the UK had the lowest ex-factory and public prices in the EU-5.

Our study however showed that looking at average annual treatment cost ratios offered a restrictive view as the orphan drug cost ratios were greatly heterogeneous. This has been observed to be true in all the countries analysed. The minimum and maximum ratios ran from 0.26 to 1.92 in all countries which means some orphan drugs are 74% cheaper than the UK and some are nearly twice the price of the UK counterpart. The mean and median cost ratios were interesting in their own right but showed limited insight. When we assessed the proportion of orphan drugs under four cost ratio categories (Figure 2), a relevant number of orphan drugs were cheaper and a relevant number were more expensive than the UK in all countries, except Norway where 90% of the treatment costs were cheaper than the UK. These proportions did not correlate perfectly with the average annual treatment cost ratios and thus it can be interpreted that the average cost ratios simplified the analysis and did not reflect reality. In Spain, although the average annual treatment costs ratio showed it was overall more expensive than the UK by 7% (ratio 1.07), 55% of orphan drugs were cheaper than the UK and 30% were >10% cheaper. In Sweden, although the averaged costs ratio showed it was overall cheaper than the UK by 1% (ratio of 0.99), 51% were more expensive than the UK and 29% were >10% more expensive than the UK counterparts.

The heterogeneity of prices may be due to multiple factors. Pricing of orphan drugs is a complex process with multiple price determinants. Two recent studies looked into the decision drivers of orphan drugs price setting in Europe and both supported the general notion that orphan drug pricing in Europe is inconsistent and non-transparent. Onakpoya et al. [33] in 2014 showed that the annual cost of drugs in the UK did not appear to be related to their clinical effectiveness and that there is no clear and standardised mechanism for determining their prices. The difficulties in generating evidence for rare diseases and the lack of robust information when the price is set are probable factors. Picavet et al. [25] in 2014 through a multiple regression analysis showed that prices of orphan drugs in six EU countries are influenced by factors such as the availability of an alternative drug treatment, repurposing of the drug, the length of treatment, the administration route, the presence of multiple indications, and the impact in overall survival and quality of life (QoL). No association was found between annual treatment cost of orphan drugs across countries and the different pricing and reimbursement systems. The study however indicates that significant vagueness still surrounds the orphan drug pricing mechanism.

HTAs generally rely on clinical and economic evidence upon launch. Additionally, societal preferences and social value judgments are sometimes considered in the deliberative process and are critical in drug appraisal, especially for rare diseases which have limited clinical evidence, high uncertainty, and a high unmet need. A study by Nicod et al. [34] in 2016 did a qualitative themative analysis of the scientific and social value judgments in HTAs for 10 orphan drugs in England, Scotland, France, and Sweden. Beyond the assessment of clinical and economic evidence, 125 ‘other considerations’ were identified (e.g. national priorities such as a rare disease plan; nature of the disease affecting the patient, rarity, orphan status, small population, unmet need, type of treatment benefit (curative vs. symptomatic), and innovativeness of treatment). Depending on the HTA body, 18–100% of these were found to be decision drivers during appraisals and some were found to be non-quantified or non-elicited and pertained to the assessor’s value judgment. The differences in considering soft information, on how and to what extent they inform the deliberative process, and the respective weights placed for each decision may be important factors leading to the price differences of orphan drugs within and between countries.

The inconsistencies in orphan drug pricing in Europe as presented in Table 3 were validated in this study when we assessed the cost ratios per disease areas. The average cost ratios were heterogeneous across the eight disease areas, in all countries (Figure 4). The variation lessened when we only analysed the top three diseases with the most available orphan drug prices in the UK. As we have seen above that averaged ratios may be limiting, heterogeneity may be expected to be seen within each top disease area.

**Table 3.** Available literature on orphan drug prices in the EU.

Publication	Year	Geographical scope	N of OD analysed	Methodology	Results
Alcimed [31]	2004	EU-25	10	For each OD, the manufacturer’s price before taxes (MPBT, ex-factory) was compared to the lowest in all countries and the ratios were averaged by country.	The maximum variations between countries are not very different, on average 122% of the lowest price (105–173%).
					Germany and France had the highest prices.
					Spain, Portugal, and the UK had the lowest prices.
				For each OD, the public price including taxes (PPIT) were used to compute for annual cost per patient and country comparison done.	The PPIT cost variations were greater than MBPT (above), and may reach 70%.
					Austria has the highest annual cost per patient.
					Followed by Germany and Denmark.
					Then, Finland, France, Ireland.
					Then, Spain, Portugal, UK and Sweden.
Eurordis [5]	2010	10: Belgium, Denmark, France, Greece, Hungary, Italy, The Netherlands, Romania, Spain, Sweden	60	For each OD, the ‘national price’* was compared to the lowest in the 10 countries and the ratios were averaged by country.	Italy: 150–160%
					Denmark and Greece: 140–150%
					Hungary and Spain: 110–120%
					Belgium, France, and Romania:100–110%
				For each OD, the ‘national price’ was compared to the mean value in the 10 countries and the ratios were averaged by country.	Italy: 120–130%
					Denmark and Greece: 110–120%
					Hungary and Spain: 90–100%
					Belgium, France, and Romania: 80–90%
Macarthur, D [32]	2011	7: France, Germany, Italy, Spain, Sweden, Switzerland, UK	60	Direct comparison of public prices.	When Italian price was available (N = 45), it was highest in the EU-5 except for three drugs.
					When UK price was available (N = 56), it was the cheapest in the EU-5 except for eight cases.
			12	Direct comparison of ex-factory price (estimated by deducting distribution margins and VAT from public price).	Germany tended to have the highest ex-factory prices in the EU-5.
					UK had most of the lowest ex-factory prices. Some more recent drugs had higher prices.
					Swiss prices were higher than in Germany.

*‘National price’ was not defined

A report in 2011 by the European Parliament [35] referenced the same price heterogeneity in non-orphan in-patent pharmaceuticals in European countries. When comparing the five largest EU pharmaceutical markets (France, Germany, Italy, Spain, and UK), Germany had the highest retail prices for patented drugs (23% higher than the average of the five countries), followed by the UK (exactly at the five-country average), Spain (5% lower), Italy (6% lower) and France (14% lower). Differences were however shown to be greater when considering individual pharmaceuticals. When seven in-patent prescription oncology drugs were selected, significant price variability exists across countries and across products, with the greatest price difference of 50–60%. No country demonstrated consistently higher prices for all products. Further evidence was provided for a range of widely used older drugs, where price differences were significantly higher, with the greatest difference of four to one. These analyses were done prior to 2010 and it was noted that despite the significant price differences, price differences among member states have been decreasing due to the mechanism of external reference pricing (ERP), which has become the most common price setting measure for pharmaceuticals in EU member states. ERP, which is also known under different names such as external price referencing (EPR) or international price comparison/benchmarking, is defined as ‘the practice of using the price(s) of a medicine in one or several countries in order to derive a benchmark or reference price for the purposes of setting or negotiating the price of the product in a given country’ (p. 5).[36] No objective and comprehensive literature has found to what extent ERP is used in orphan drug pricing in Europe. In the midst of a lack of European consensus of how orphan drugs should be treated in terms of pricing and market access,[4,17,22] collaborations have been pursued to give more insight into pricing and reimbursement decisions for orphan drugs.[18] Multiple criteria decision analysis (MCDA) frameworks which incorporate relevant value elements into P&R decision in a transparent and consistent matter [37–40] and other price control mechanisms such as cost-plus or rate of return models employing yardsticked cost allocations and rate of return calculations in setting orphan drug prices have been proposed.[41]

When we divided the UK orphan drugs into four quartiles based on the most expensive and least expensive, the averaged ratios among the four quartiles varied minimally in all countries except France. This shows that the cost differences were similar for the most expensive and least expensive orphan drugs in the UK, and all orphan drugs in between (Figure 3). In France, the variation showed that orphan drugs costs in France were on average 9% cheaper than cheapest treatment costs in the UK (quartile 4) but were 15% more expensive than the highest treatment costs in the UK (quartile 1). These variations could be explained by the pricing process in France which is driven by the evidence of additional benefit over the next best alternative. If the evidence is not compelling, the product will be requested to offer a discount over the comparator that may be a cheap product. On the other hand, if the product has shown evidence of additional benefit acknowledged by the HTA agency, it will be allowed free pricing with a cap at the level of the EU big four prices.

This analysis showed that orphan drug prices vary across and within European countries and reaffirms that there is currently no European consensus on how the value of orphan drugs are and should be assessed.[19] Indeed, in the literature, orphan drugs pricing in Europe have been referred to as the ‘black box’.[22,23] This does not seem to come as a surprise as there are no specific pathways for market access and price setting of orphan drugs in these countries.

Based on the finding of this analysis, we may not be able to predict which country will have higher or lower prices for orphan drugs based on two rationales. First, the price difference was quite small across countries when we looked at the average treatment cost ratios. Secondly, the price differences of orphan drugs within each country were greatly heterogeneous when we looked at the cost ranges and the proportion of orphan drugs cheaper or more expensive than the UK. Thus, no country may be considered as one which allows higher orphan drug prices or imposes lower prices.

Only three publications related to the comparison of EU prices in rare diseases were found in the literature (Table 3). These studies are relatively less comprehensive in terms of the number of orphan drugs analysed. They date from 2004–2011 when fewer orphan drugs were launched. In all three studies, the methodologies used consisted of directly comparing list prices of comparable drugs and packages. In our study, annual treatment costs were used to compare prices. Comparing prices directly from the database is limiting as it only provides prices per available pack and different packaging exists per country. To our knowledge this work is currently the most comprehensive research comparing orphan drug treatment costs in Europe.

### Limitations of this research

The prices of the drugs are the listed prices which are often not aligned with the actual net prices, a better reflection of healthcare expenditures on orphan drugs. Confidential discounts, rebates, price volume agreements, cap expenditures, and tenders may be negotiated at the national, regional, or healthcare provider level, which may distort the ex-factory price. However, the potential discounts and rebates are expected to be reasonably homogeneous for all orphan drugs within the same country.

The cost ratios presented here only used the UK as a reference and did not present cost differences between the other six countries. Other combinations are possible which may give specific insights although the sample sizes are smaller. These are provided in Appendix C to Appendix H (Figures 6–35)  in the supplementary file.

It is of note is that price is not a good proxy for drug usage. As an example, obtaining a drug price may be relatively easy in the UK but it may not guarantee patient access, and is only a formal step. A manufacturer may obtain a high price but no market uptake across the whole of the UK. Further research on the relationship of orphan drug price and market share in value and volume will provide additional insight.

Furthermore, given the differences among countries in their ability and willingness to pay for healthcare, further analysis is needed to assess the differences or similarities in GDP, PPP, and healthcare expenditure as part of GDP and their relationship with orphan drug prices. A between-country comparison will validate that the patterns of price differences and access are not only due to differences in willingness to pay and/or economic situation and are attributable to local pricing policies and negotiations.

Finally, while the comparison of prices in the Eurozone may be more stable, countries outside the Eurozone, such as the UK (GBP), Norway (NOK), and Sweden (SEK) may have experienced substantial changes in the exchange rates of their currency during our period of interest. Thus, the exchange rates may have affected the prices in a way that is not related to the value assessment. In this study, the earliest price assessed in the UK is dated 1 June 2002, in Norway 1 June 2008, and in Sweden 1 March 2004. Conversion of prices to Euros was done on 14 June 2016 using country-specific exchange rates of the same date. The average Euro/GBP exchange was 0.63 in 2002, was stable until 2007, peaked to 0.89 in 2009, dropped to 0.73 in 2015 and ended at 0.80 in 2016.[42] The average Euro/NOK exchange was 8.23 in 2008, dropped to 7.48 in 2012, and peaked to 9.38 in 2016.[42] The average Euro/SEK exchange was 9.13 in 2004, was stable until 2007, peaked to 10.63 in 2009, dropped to 8.66 in 2013, ending at 9.37 in 2016.[42] During the study period, the GBP has been erratic but with a general upward trend. The NOK’s value is increasing against the Euro and SEK has been fairly stable. These changes are not seen to invalidate the findings of this study. Additionally, the same conversion rate per country was used for all price and cost calculations. Figures of the currency trends over the studied period are provided in Appendix B in the supplementary file.

## Conclusion

This study shows that compared to the UK, the average annual treatment costs of orphan drugs were more expensive in France, Germany, Italy, Spain, and were cheaper in Sweden and Norway, with minimal variation. The average and median cost differences (cost ratios) however provided limited insight as the cost differences showed great heterogeneity in all countries, showing that orphan drug prices vary widely across and within European countries. This study suggests that in Europe, we may not be able predict which country may have higher or lower prices for orphan drugs. Further studies looking at the determinants of orphan drug prices in Europe may provide insight if there are specific drivers for orphan drug pricing per country.

## Supplementary Material

Supplemental Data
