# Supplementary material for: A comparative study of orphan drug prices in Europe
Source: J Mark Access Health Policy. 2017 Mar 29;5(1):1297886. doi: 10.1080/20016689.2017.1297886 (PMC5405561; doi:10.1080/20016689.2017.1297886)
Supplement: Supplemental Data [file zjma_a_1297886_sm9019.docx]

Appendix (Supplementary File)

## Appendix A. Drugs used in the analysis with corresponding indication

| Drug | Indication simplified |
| --- | --- |
| Adcetris | Hodgkin Disease |
| Adempas | Pulmonary hypertension |
| Afinitor* | Renal cell carcinoma |
| Aldurazyme* | Mucopolysaccharidosis I |
| Alprolix** | Haemophilia B |
| Arzerra | Chronic lymphocytic leukaemia |
| Atriance | Precursor T-Cell lymphoblastic leukemia-lymphoma |
| Blincyto | Precursor Cell lymphoblastic leukemia-lymphoma |
| Bosulif | Chronic myeloid leukaemia |
| Bronchitol | Cystic fibrosis |
| Busilvex* | Conditioning treatment prior to haematopoietic-progenitor-cell transplantation |
| Carbaglu | N-acetylglutamate synthetase deficiency |
| Cayston | Gram negative bacterial lung infection in cystic fibrosis |
| Ceplene | Acute myeloid leukaemia |
| Cerdelga | Gaucher disease type 1 |
| Coagadex** | Hereditary factor X deficiency |
| Cometriq | Metastatic medullary thyroid carcinoma |
| Cresemba | Mucormycosis |
| Cyramza* | Stomach neoplasms (Gastric cancer) |
| Cystadane | Homocystinuria |
| Dacogen | Acute myeloid leukaemia |
| Darzalex** | Plasma cell myeloma |
| Defitelio** | Severe hepatic veno-occlusive disease |
| Deltyba | Tuberculosis |
| Diacomit | Severe myoclonic epilepsy in infancy |
| Elaprase | Mucopolysaccharidosis II |
| Esbriet | Idiopathic pulmonary fibrosis |
| Evoltra | Precursor cell lymphoblastic leukemia-lymphoma |
| Exjade | Iron overload |
| Fabrazyme* | Fabry disease |
| Farydak | Multiple myeloma |
| Firazyr | Hereditary angioedemas |
| Firdapse | Lambert-Eaton myasthenic syndrome |
| Galafold** | Fabry disease |
| Gazyvaro | Chronic lymphocytic leukaemia (follicular lymphoma) |
| Gliolan | Malignant glioma |
| Glivec* | Chronic myeloid leukaemia |
| Glybera | Familial lipoprotein lipase deficiency |
| Granupas | Tuberculosis |
| Hetlioz** | Non-24-hour sleep-wake disorder |
| Holoclar** | Limbal stem cell deficiency |
| Iclusig | Chronic myeloid leukaemia |
| Idelvion | Haemophilia B |
| Ilaris* | Cryopirin associated syndromes |
| Imbruvica | Mantle cell lymphoma |
| Imnovid | Multiple myeloma |
| Increlex | Laron syndrome |
| Inovelon | Epilepsy |
| Jakavi* | Chronic idiopathic myelofibrosis |
| Kalydeco | Cystic fibrosis |
| Kanuma | Lysosomal acid lipase deficiency |
| Ketoconazole HRA | Endogenous Cushing’s syndrome |
| Kolbam | Inborn errors in primary bile acid synthesis |
| Kuvan | Phenylketonurias |
| Kyprolis | Multiple myeloma |
| Lenvima | Differentiated thyroid carcinoma |
| Litak* | Hairy cell leukemia |
| Lynparza | Ovarian cancer |
| Lysodren* | Adrenal cortex neoplasms |
| Mepact | Osteosarcoma |
| Mozobil | Hematopoietic stem cell transplantation for Multiple myeloma, Lymphoma |
| Myozyme | Glycogen storage disease type II |
| Naglazyme* | Mucopolysaccharidosis VI |
| Nexavar | Renal cell carcinoma |
| Nexobrid | Deep partial- and full-thickness thermal burns |
| Nplate | Thrombocytopenic idiopathic purpura |
| Ofev | Idiopathic pulmonary fibrosis |
| Opsumit | Pulmonary arterial hypertension |
| Orfadin* | Tyrosinemias |
| Orphacol | Digestive system diseases, Inborn errors of metabolism |
| Pedea* | Patent ductus arteriosus |
| Peyona | Primary apnoea |
| Photobarr* | Barrett esophagus |
| Plenadren | Adrenal insufficiency |
| Prialt* | Pain injections, spinal |
| Procysbi | Nephropathic cystinosis |
| Raxone | Leber’s hereditary optic neuropathy |
| Ravicti** | Carbamoyl-phosphate synthase-1 deficiency |
| Replagal* | Fabry disease |
| Revatio* | Pulmonary hypertension |
| Revestive | Short-bowel syndrome |
| Revlimid | Multiple myeloma |
| Revolade* | Idiopathic thrombocytopenic purpura |
| Savene | Extravasation of diagnostic and therapeutic Materials |
| Scenesse** | Erythropoietic protoporphyria |
| Signifor | Cushing’s disease |
| Siklos | Sickle cell anemia |
| Sirturo | Pulmonary multidrug resistant tuberculosis |
| Soliris | Paroxysmal nocturnal haemoglobinuria |
| Somavert | Acromegaly |
| Sprycel | Chronic myelogenous leukemia, BCR-ABL positive |
| Strensiq | Hypophosphatasia |
| Strimvelis | Adenosine deaminase deficiency |
| Sutent* | Malignant gastrointestinal stromal tumours |
| Sylvant | Multicentric Castlemans disease who are human immunodeficiency virus (HIV) negative and human herpesvirus-8 (HHV-8) negative |
| Tasigna | Chronic myelogenous leukemia, BCR-ABL positive |
| Tepadina | Conditioning treatment prior to allogeneic or autologous haematopoietic progenitor cell transplantation (HPCT) in haematological diseases |
| Thalidomide-Celgene | Multiple myeloma |
| Thelin | Pulmonary arterial hypertension |
| Tobi Podhaler | Pseudomonas aeruginosa lung infection in cystic fibrosis |
| Torisel | Renal cell carcinoma |
| Tracleer* | Pulmonary arterial hypertension and Chronic thromboembolic pulmonary hypertension |
| Translarna** | Duchenne muscular dystrophy |
| Trisenox* | Acute promyelocytic leukaemia |
| Unituxin** | Neuroblastoma |
| Uptravi | Pulmonary arterial hypertension |
| Ventavis* | Primary pulmonary hypertension |
| Vidaza | Myelodysplastic syndromes |
| Vimizim | Mucopolysaccharidosis, type IVA (Morquio A Syndrome) |
| Volibris | Pulmonary arterial hypertension and chronic thromboembolic pulmonary hypertension |
| Votubia | Renal angiomyolipoma with tuberous sclerosis complex |
| VPRIV | Gaucher disease |
| Vyndaqel | Transthyretin amyloidosis |
| Wakix** | Narcolepsy |
| Wilzin* | Hepatolenticular degeneration |
| Xagrid | Essential thrombocythemia |
| Xaluprine | Acute lymphoblastic leukaemia |
| Xyrem* | Narcolepsy |
| Yondelis | Sarcoma |
| Zavesca | Gaucher Disease |

*Orphan designation withdrawn or expired

** No prices available in the countries in scope; probably not commercially available

## Appendix B. Currency Exchange Rate Trends for UK, Norway and Sweden

## Appendix C: Comparison of the annual treatment cost of comparable drugs to reference country: France

**Figure 6. Mean, median, minimum, maximum ratios per country using France as reference**

**Figure 7. Percentage of orphan drugs (OD) under 4 cost ratio categories using France as reference**

**Figure 8. Averaged ratios when France reference list was divided into 4 quartiles based on France costs. Quartile 1 includes orphan drugs with the highest annual treatment costs in France and quartile 4 includes orphan drugs with the cheapest annual treatment costs in France**

**Figure 9. Averaged ratios when France reference list was divided into 9 disease areas**

**Figure 10. Averaged ratios for top 3 disease areas with most orphan drugs available in France**

## Appendix D: Comparison of the annual treatment cost of comparable drugs to reference country: Germany

**Figure 11. Mean, median, minimum, maximum ratios per country using Germany as reference**

**Figure 12. Percentage of orphan drugs (OD) under 4 cost ratio categories using Germany as reference**

**Figure 13. Averaged ratios when Germany reference list was divided into 4 quartiles based on Germany costs. Quartile 1 includes orphan drugs with the highest annual treatment costs in Germany and quartile 4 includes orphan drugs with the cheapest annual treatment costs in Germany**

**Figure 14. Averaged ratios when Germany reference list was divided into 9 disease areas**

**Figure 15. Averaged ratios for top 3 disease areas with most orphan drugs available in Germany**

## Appendix E: Comparison of the annual treatment cost of comparable drugs to reference country: Italy

**Figure 16. Mean, median, minimum, maximum ratios per country using Italy as reference**

**Figure 17. Percentage of orphan drugs (OD) under 4 cost ratio categories using Italy as reference**

**Figure 18. Averaged ratios when Italy reference list was divided into 4 quartiles based on Italy costs. Quartile 1 includes orphan drugs with the highest annual treatment costs in Italy and quartile 4 includes orphan drugs with the cheapest annual treatment costs in Italy**

**Figure 19. Averaged ratios when Italy reference list was divided into 10 disease areas**

**Figure 20. Averaged ratios for top 3 disease areas with most orphan drugs available in Italy**

## Appendix F: Comparison of the annual treatment cost of comparable drugs to reference country: Spain

**Figure 21. Mean, median, minimum, maximum ratios per country using Spain as reference**

**Figure 22. Percentage of orphan drugs (OD) under 4 cost ratio categories using Spain as reference**

**Figure 23. Averaged ratios when Spain reference list was divided into 4 quartiles based on Spain costs. Quartile 1 includes orphan drugs with the highest annual treatment costs in Spain and quartile 4 includes orphan drugs with the cheapest annual treatment costs in Spain**

**Figure 24. Averaged ratios when Spain reference list was divided into 7 disease areas**

**Figure 25. Averaged ratios for top 3 disease areas with most orphan drugs available in Spain**

## Appendix G: Comparison of the annual treatment cost of comparable drugs to reference country: Norway

**Figure 26. Mean, median, minimum, maximum ratios per country using Norway as reference**

**Figure 27. Percentage of orphan drugs (OD) under 4 cost ratio categories using Norway as reference**

**Figure 28. Averaged ratios when Norway reference list was divided into 4 quartiles based on Norway costs. Quartile 1 includes orphan drugs with the highest annual treatment costs in Norway and quartile 4 includes orphan drugs with the cheapest annual treatment costs in Norway**

**Figure 29. Averaged ratios when Norway reference list was divided into 10 disease areas**

**Figure 30. Averaged ratios for top 3 disease areas with most orphan drugs available in Norway**

## Appendix H: Comparison of the annual treatment cost of comparable drugs to reference country: Sweden

**Figure 31. Mean, median, minimum, maximum ratios per country using Sweden as reference**

**Figure 32. Percentage of orphan drugs (OD) under 4 cost ratio categories using Sweden as reference**

**Figure 33. Averaged ratios when Sweden reference list was divided into 4 quartiles based on Sweden costs. Quartile 1 includes orphan drugs with the highest annual treatment costs in Sweden and quartile 4 includes orphan drugs with the cheapest annual treatment costs in Sweden**

**Figure 34. Averaged ratios when Sweden reference list was divided into 6 disease areas**

**Figure 35. Averaged ratios for top 3 disease areas with most orphan drugs available in Sweden**
